# Supplementary material for: Metascientific replication project with the advanced meta-experimental protocol of the transparent psi project procedures for testing the precognitive effect claimed by Bem
Source: PLoS One. 2025 Nov 5;20(11):e0335330. doi: 10.1371/journal.pone.0335330 (PMC12588471; doi:10.1371/journal.pone.0335330)
Supplement: Attachment — Submitted filename: Review.pdf [file pone.0335330.s001.pdf]

Review

The manuscript, “Metascientific replication project with the advanced meta-experimental protocol of the transparent psi project procedures for testing the precognitive effect claimed by Bem,” investigates the replicability of claims about precognitive effects reported by Bem (2011) using advanced methodologies, including the AMP-TPP protocol, across three large-scale studies. While Study 2 replicated an exploratory finding suggesting a slight below-chance effect, neither the original precognitive effect nor the replicated anomaly was confirmed in Study 3. The results highlight methodological advancements for rigor in psychological research and suggest that observed results are better explained by method-derived anomalies rather than psi-theory.

The manuscript features a well-powered design and demonstrates commendable adherence to open-science and transparency practices, significantly enhancing its credibility and reproducibility while offering well-founded criticism of psi-theory. However, several areas warrant improvement, including clarifying key methodological details, improving the comprehensibility of the discussion, and addressing the results more thoroughly with critical consideration of specific psi concepts. Overall, this study is a valuable contribution to the field, and the authors’ commitment to rigorous research practices is evident. I believe that addressing the issues outlined above in a major revision would greatly enhance the manuscript’s quality.

For detailed comments, see below.

Abstract

The discussion section provides a commendable critique of the interpretation of psi-theory. However, the abstract could better reflect these critical insights. Currently, it presents a psi-derived anomaly (lines 43-44) as a plausible explanation for anomalous results, which risks implying equivalence with the much more scientifically grounded explanation of a method-derived anomaly. I recommend revising the abstract to highlight the significant limitations of psi-theory as an explanation, drawing on the critical insights from the discussion section.

Introduction

1) Consider avoiding the introduction of abbreviations in headings, such as 'TPP' in '1.3.1. The Transparent Psi Project (TPP) Procedures.' Instead, use the full term, e.g., 'Procedures for the Transparent Psi Project,' and introduce the abbreviation in the main text of the section.

2) The manuscript employs the term “counterfactual” multiple times, but it may be unclear to readers who are not already familiar with its usage in this field. Although the preregistration provides clarification, the main text currently does not. Please offer a concise definition of “counterfactual” in the manuscript itself—ideally at first mention—to ensure all readers can fully grasp its relevance and application in the study.

3) Lines 132-133: The text states that the anomalous effect was observed only when erotic images were used as a reward, but it is unclear how these images functioned as such. Were they only revealed to participants upon correct guesses, or was there another mechanism at play?

Although this is explained later in the manuscript, it would greatly help readers to have a concise explanation of Bem's original design and the reward structure at this point in the text. This also applies to the section covering lines 212–220.

4) Lines 166–171: The three classes of errors—class-A, class-B, and class-C—are presented in a rather abstract manner, making them difficult to understand without concrete examples. Additionally, the phrase "repeated performances" in the discussion of class-B errors is unclear; please clarify what exactly is meant by "performance" in this context. It would be beneficial to offer a clearer definition of each error category earlier in the manuscript, for instance in Section 1.3.2, so that readers are not left wondering about these distinctions until Section 2.2.2.

5) "For explanation, a hidden bias might produce a false-negative result because of a concealed shift of the measurement baseline as a function of a falsely reduced, or falsely increased, rate of successful guesses which might—in turn—prohibit the observation of a true-positive finding" (lines 197–200). This explanation is somewhat convoluted and unclear. What is meant by "a concealed shift of the measurement baseline"? I think the word baseline could be misleading in this context, as you refer to the chance level, if I am not mistaken. Maybe you can clarify this and make it a little bit more comprehensive.

6) The sentence *"For a brief review, Bem [35] reported that a person's precognitive ability (the independent variable) was revealed as an effect on the percentage of successful guesses in the selection of the correct target side (left or right) on a computer screen (the dependent variable) where an erotic image will be presented as a reward experience (the moderator variable)."* (l. 212-216) is quite lengthy and hard to follow. You might consider revising it for brevity and clarity, ensuring each variable is clearly introduced and its role in the study is made more comprehensible.

7) Lines 222–225: This explanation of sham-erotic trials is unclear. If these trials involve no image being displayed, it is difficult to understand how a "successful" guess is determined and what happens in place of the erotic image. Please clarify what participants see on the screen during these sham-erotic trials. Again, I realize that you explain this in more detail later on, but as with previous comments, I believe it would help the reader to fully understand the sham conditions already at this stage.

8) Lines 229–248: This section provides a solid explanation of the control strategy for participants; however, it contains some redundancies and could benefit from improved language flow. I recommend revising this section to address these issues and enhance overall clarity and readability.

9) Lines 255–257: What is the specific advantage of using a Random Event Generator as a control variable? Wouldn't a binomial test with p=0.5 effectively account for random selection, rendering the Random Event Generator unnecessary? Please clarify and emphasize the added

value of simulating random choice with a machine compared to simply testing a binomial function.

Methods

10) Sample Size Estimation: The manuscript does not provide information on how the sample size was determined. While the preregistration includes a section describing Monte Carlo Simulation Studies, it does not specify the assumptions underlying these simulations. The preregistration also cites a preprint by Kekecs et al. (2019), where the sample size calculation is thoroughly described. However, to ensure readers are adequately informed, a brief explanation of the sample size justification should also be included in the main manuscript.

Additionally, the authors should provide more detail on the rationale for increasing the sample size from one experiment to the next. This explanation would help readers better understand the methodological decisions and their implications.

11) Please include details about the experimental software that was used in the experimental setup.

12) Section 2.2.2: How does an S(O) trial differ from an S(X) trial? If I understood the sham trials correctly (and I am not entirely sure that I did), these trials seem to conclude without showing any picture (whether erotic or non-erotic) to the participant, even if they guessed the side of the image (or non-image?) correctly. However, this raises a question: how does the absence of an erotic picture differ from the absence of a non-erotic picture? Aren't they effectively the same? While you address this later in the manuscript, a brief clarification earlier in the text would prevent readers from becoming confused or wondering about this distinction.

13) Lines 404–412: The terms "X" and "O" are used in this section but are only explained later in the manuscript, and even then somewhat implicitly. To ensure that readers can fully understand the meaning of this nomenclature, consider providing a clear and concise explanation at the first mention of these terms. This will help avoid confusion and improve the overall clarity of the manuscript.

14) Lines 426–444: This section contains numerous redundancies and over-explains the concept of pure sessions and mixed sessions, with repeated references to Figure 3. To improve readability and streamline the text, consider consolidating the explanations and minimizing repetitive references to the figure, focusing on delivering the essential information succinctly.

15) Lines 439–444: Assuming the existence of a psi-phenomenon or a similar concept, wouldn't informing participants that their choice was wrong—when it was actually correct—risk inducing frustration? This could open the study to criticism from psi-supporters, who might argue that the presence of sham trials does more than control for the absence or presence of erotic image rewards; it could actively introduce frustration. A psi-defender might suggest that such

frustration could interfere with psi ability. Would it not have been better to provide participants with feedback about whether their choices were correct or incorrect without displaying the image? This approach could control for reward absence while avoiding potential frustration effects. This potential limitation should be addressed and discussed in the Discussion section.

16) Lines 450–456: The detailed description of the consent procedure is overly lengthy and could be streamlined. Condensing this section would improve readability without compromising the essential information about the consent process.

17) Lines 462–465: The number of participants is missing from this section. Including study-wise reporting of participant demographics would enhance transparency and allow readers to better understand the sample characteristics for each study.

18) Section 2.6.1: It seems there were no exclusion criteria for data in the experiment. If that is the case, please state this explicitly to ensure clarity for the reader.

19) Lines 496–497: The statement *"Data was pooled across participants for each experimental condition of each study"* is unclear. Please clarify what is meant by that statement.

20) Line 552: What does BUJ stand for?

21) Line 558: You state, "Based on this, a bidirectional analysis was conducted (...)." Why is a bidirectional analysis conducted when the hypothesis being tested is unidirectional—that the hit rate is below 50%? Please clarify the reasoning behind this choice of analysis, as it seems inconsistent with the stated hypothesis.

22) Section 2.6.7: The procedures for Experiments 2 and 3 are heavily influenced by the results of Experiment 1, particularly as these results motivated the analyses to replicate a systematic deviation of the hit rate below 50%. Presenting the methods for all experiments in direct succession makes it challenging to follow the reasoning and motivations behind certain design choices, such as the analysis of temporal dynamics of hit rates described in lines 594–596. To improve clarity, I strongly recommend presenting the results of Experiment 1, followed by an intermediate (brief) discussion of Study 1, before introducing the methods for the subsequent experiments. This approach would help readers better understand the rationale for specific design and analysis choices.

23) Lines 594–605: The exploratory analysis of the temporal dynamics of hit rates is somewhat puzzling. It seems odd to describe the plot elements intended to visually investigate these dynamics (lines 599–605) without including such a plot in the manuscript. Additionally, it is unclear on what values the plotted 95% confidence intervals are based. Please revise this

section to clarify exactly what is being plotted and consider including the relevant plot(s). If multiple plots were generated and could not fit within the manuscript, this should be explicitly stated in this section to prevent readers from wondering why these plots are absent.

24) Lines 632–633: The statement, "this reporting format was chosen because it is the most common in the field of psychology and parapsychology," is debatable. While ±SE error bars may be common, confidence intervals (CIs) typically provide more information regarding the interpretability and significance of effects. I am a bit confused as to why you opted to depict the SE rather than the CI, although using SE is acceptable.

25) Line 655: The statement, *"All preregistered analyses, frequentist, and Bayesian confirmed model* M_0*,"* is problematic. Frequentist analyses cannot confirm M_0 in the way suggested here; they can only fail to reject the null hypothesis. In other words, the absence of evidence is not evidence of absence in the frequentist framework. Please revise this phrasing to reflect the limitations of frequentist analyses accurately. However, Bayesian analyses can indeed provide evidence supporting M_0, so this distinction should be made clear here and in other relevant sections of the manuscript.

26) Line 669: Revise *"The Bayes Factor (0, 1)"* to ensure consistency with the earlier use of BF_{01}, which is more common and aligns with the manuscript's established nomenclature. Please ensure this is consistent throughout the text.

27) Lines 687–689: You only provide a verbal description of finding null results in this section. To ensure transparency and completeness, please report the full results, including numerical values, directly in the text. Avoid relying on the figure caption for this information, as including it in the text section will enhance clarity and accessibility for readers.

28) Lines 711–712: The phrasing "this apparent 0.5% effect" is unclear. Please rephrase to make it more comprehensive.

29) Lines 722–724: The statement *"These null results seemed to confirm the finding reported by Bem [35] that neutral trials, i.e., trials with non-erotic images, do not lend themselves to precognition"* is somewhat misleading, as your well-powered study also found no evidence of precognition with erotic images. Please rephrase this to emphasize the absence of any evidence for general precognition in this study, avoiding implications that precognition might still occur with erotic images.

30) Lines 724–726: The statement *"found a null result"* is misleading, as it suggests an equivalence test was conducted. Since no equivalence test was performed, you can only state that no significant effect of precognition was found. Additionally, "only for both erotic trials (X) and sham-erotic trials (S(X))" implies a significant effect for the mixed-neutral (O) condition, but

Fig. 6 shows no significant p-value (p = 0.185). It seems this is inferred from the mixed-O error bar not crossing the 50% line. However, the error bar represents SE, not the 97.5%-CI, so this conclusion is incorrect. Please correct the statement or clarify what you mean by it. Also consider using proper CI representation instead of SE, as suggested earlier.

Discussion

31) I may have missed it, but the Discussion section does not seem to explicitly address how the replicated effect in Study 2, which occurred in the *opposite direction* of the assumed precognition effect, could be interpreted as evidence for psi-based precognition. Why would participants be systematically wrong in predicting the appearance of erotic stimuli? Even setting aside the valid criticisms of psi-based explanations raised in the discussion, does this reversed effect provide any meaningful evidence for psi phenomena? This question warrants clarification.

32) The discussion effectively balances the rigorous scientific perspective with the psi-based perspective when interpreting the results. You also provide a thorough and commendable critique of psi-based approaches. However, the discussion is occasionally overly extensive and, at times, redundant. Certain sections are difficult to follow and could benefit from clarification (see specific suggestions below).

33) Line 899: "(e.g., [13, 42-47)" Missing closing square bracket in citation

34) Line 925: The terms *"trickster effect," "psi-missing,"* and *"experimenter psi"* are introduced without sufficient explanation, leaving readers unclear about how these concepts might account for the results within a psi framework. Currently, these terms are mentioned without connecting them meaningfully to the findings. A more thorough discussion is needed to explain how these ideas could be applied to interpret the results.

35) Lines 925–927: The statement "some special, psi-conducive factor could have been missing in the present study and—therefore—the original psi effect of Bem [35] could not be replicated" does not address the surprising opposite direction of the effect observed in Studies 1 and 2. How would a psi-follower explain (their way out of) this reversed effect? If the failure to replicate Bem's findings is attributed to a missing "psi-conducive" factor, this explanation does not account for why participants performed systematically worse than chance rather than at chance. If psi-proponents cannot provide a plausible explanation for this, it should be explicitly addressed, as it further reinforces criticisms that psi lacks a rigorous, falsifiable theoretical framework.

36) Lines 930–944: In this section, you discuss the high stakes of accepting psi explanations, noting the significant challenge they pose to conventional assumptions. Given these high stakes, one could argue for the adoption of a more rigorous alpha threshold in psi research. A

false-positive result—such as the one likely observed in Study 2—has a much higher "cost" than a false-negative result. For instance, accepting a false-positive might mean we'd have to start firing physics professors en masse. Adopting a stricter alpha threshold, as suggested in [https://www.nature.com/articles/s41562-018-0311-x], could help mitigate such risks and improve the reliability of findings in this controversial area of research.

37) Lines 960–969: This section is convoluted and difficult to follow. Please clarify for better readability.

38) Section 4.4: The heading *"On observed anomalous results in parapsychology as ULPs"* is somewhat unclear and took me a moment to understand that this section discusses how psi-ists interpret ULPs as psi-related phenomena. Consider changing the title to something like *"Psi-Explanation Attempts of ULPs"* for greater clarity.

39) Fig. 10: I don't think this figure is essential and could be removed.

40) Lines 1038–1044: The terms "Ψ-epistemic" and "Ψ-ontic interpretations" should be briefly explained for clarity. Additionally, it should be made explicit that these refer to a different kind of psi than the one assumed in precognition. Furthermore, the phrase "nonlocal ontology or quantum mechanics based on time-reversing, retrocausal ontologies" is difficult to understand for readers who are not familiar with quantum physics. Providing a brief and accessible explanation would greatly improve clarity.

41) Lines 1069–1072: Use a consistent enumeration style throughout the manuscript. For example, replace the (1), (2), ... style in this section with the previously used (i), (ii), ... style.

42) Section 4.6: This section feels somewhat redundant compared to other sections. Consider removing it and, if necessary, condensing its key points and integrating them into other relevant parts of the manuscript.

43) Lines 1110-1111: "In the history of modern science, there has been <u>no other field of research</u> that was able to maintain interest for so long as parapsychology" [underscoring added by the Reviewer for emphasis]. Is there literature supporting this claim? If so, please provide appropriate citations. Otherwise, consider softening the statement.

44) Table 1: I think this table would fit better in the results section.

45) A somewhat sacrilegious and deliberately provocative question: Psi research has been thoroughly debunked many times over, and rightly so—it's a discredited field that has repeatedly failed to demonstrate credibility. Given this, why is it still necessary to invest significant effort and resources into debunking it yet again? Couldn't this field simply be ignored as an irrelevant pursuit by a small fringe group of researchers, allowing science to focus on more pressing and meaningful topics? While I recognize that such questions might be reductive, some readers may share this perspective. Perhaps you could explicitly address these concerns in the manuscript and respond to them and also highlight how your study contributes to broader scientific and methodological progress, demonstrating its relevance beyond merely revisiting psi research.
