# Supplementary material for: Metascientific replication project with the advanced meta-experimental protocol of the transparent psi project procedures for testing the precognitive effect claimed by Bem
Source: PLoS One. 2025 Nov 5;20(11):e0335330. doi: 10.1371/journal.pone.0335330 (PMC12588471; doi:10.1371/journal.pone.0335330)
Supplement: Attachment — Submitted filename: comments on PONE-D-24-43107.pdf [file pone.0335330.s003.pdf]

General Comments:

As a statistical methodologist and meta-researcher, rather than a domain expert in psi or precognitive effects, my review focuses primarily on the methodological rigor and transparency of the submitted manuscript. I commend the authors for their ambitious effort in designing and conducting three registered replication studies to test the precognitive effect claimed by Bem (2011). The manuscript is well-written, with a clear structure and a commendable commitment to open science practices, including preregistration, data transparency, and the use of advanced methodological protocols such as the Advanced Meta-Experimental Protocol (AMP) and Transparent Psi Project (TPP) procedures. These practices align with the growing emphasis on replicability and reliability in scientific research. Plos One should be proud to consider publishing work that prioritizes such rigor, particularly in a field where replication studies remain underappreciated. The authors' efforts to address methodological challenges in psi research, such as confirmation bias and intrinsic measurement bias, are noteworthy and contribute to advancing metascience in this domain. However, I have two major concerns regarding the justification for the replication studies and the generalizability of the findings, which warrant major revisions to strengthen the manuscript.

1. The authors provide a critique of the limitations of meta-analyses in parapsychology, noting that the supportive claims for psi phenomena may be compromised by the methodological weaknesses of individual studies included in these analyses (see refs. 9, 32–34). This is a valid concern, given the prevalence of exploratory designs, lack of preregistration, and potential for questionable research practices in the field. However, the rationale for replicating Bem's Experiment 1 specifically is less robust. The authors state, "The present work investigated the most-widely discussed claim for an experimental psi effect in recent years, namely the precognitive effect claimed with Experiment 1 in the report by Bem. One major reason for choosing the paradigm of Bem Experiment 1 was the fact that a meta-analysis of 90 replication studies claimed an overall significant precognition effect—seemingly confirming the original claim of precognition". This justification is insufficiently developed, as it does not critically engage with the quality of the 90 replication studies included in Bem et al. (2016; F1000Research, 4, 1188).

Meta-analyses of replication studies, if conducted rigorously, represent high-level evidence, as they aim to synthesize findings from studies explicitly designed to test the same phenomenon, theoretically minimizing selective reporting or publication bias. Questioning the conclusions of such a meta-analysis requires a stronger empirical basis than the general methodological concerns raised in the Introduction. To strengthen their rationale, I recommend that the authors systematically assess the risk of bias in the 90 replication studies included in Bem et al. (2016). This could involve a structured evaluation using established tools (e.g., RoB 2.0) or a custom risk-of-bias framework for replication studies to examine aspects such as preregistration status, sample size adequacy, blinding, and handling of exploratory analyses. The results of this assessment should be reported as an initial section in the Results, providing a clear justification for the need for the current replication studies. Additionally, the authors could explore whether the meta-analytic conclusions depend on study quality by conducting subgroup analyses (e.g., comparing high- vs. low-

quality studies) or meta-regression to examine the influence of methodological rigor on effect sizes. Such analyses would provide a more robust foundation for the replication effort and clarify whether the claimed precognitive effect warrants further scrutiny due to methodological flaws in prior studies.

2. The authors have implemented rigorous protocols, including the AMP and TPP, to enhance the validity and transparency of their replication studies. The use of counterfactual meta-experiments (e.g., REG-based trials) and diagnostic calibration routines to detect systematic biases is particularly commendable, as these approaches address potential sources of error that are often overlooked in psychological and parapsychological research. However, the replicability of the authors' findings—particularly the anomalous below-chance effect observed in Study 2—requires verification by independent laboratories using the same experimental protocol, population characteristics, and analytical methods. The field of metascience has increasingly emphasized the value of multi-laboratory replication projects, such as Registered Replication Reports (RRRs) or Many Labs initiatives, which provide robust evidence of replicability by accounting for laboratory-specific variations and reducing the risk of idiosyncratic findings (e.g., Klein et al., 2014, Perspectives on Psychological Science). While the authors' three studies are high-powered and methodologically sophisticated, their execution within a single research framework limits the generalizability of the results.

I recommend that the authors acknowledge this limitation explicitly in the Discussion section and tone down their claims about the replicability (or lack thereof) of the precognitive effect. The authors should also discuss the feasibility of a multi-laboratory replication effort to test the robustness of their findings, particularly the unexpected below-chance effect in Study 2, which was not replicated in Study 3. This discussion should address potential sources of heterogeneity (e.g., participant characteristics, online vs. lab settings) that could influence replicability and outline how future studies could address these factors. By moderating their claims and emphasizing the need for independent replication, the authors can strengthen the manuscript's credibility and align it with best practices in metascience.

Minor comments:

While the statistical analyses are well-described, the rationale for choosing specific priors in the Bayesian proportion tests (e.g., BUJ prior, replication prior) could be clearer. The authors should briefly explain why these priors were selected and how they influence the interpretation of the Bayes factors, particularly for readers unfamiliar with Bayesian methods in parapsychology.

Please make raw data and code publicly available. There are no data and code archived in the repository provided by the authors (https://osf.io/8jteq/)

Sincerely,

Yefeng Yang. Note that I sign all my review comments
