# Supplementary material for: Metascientific replication project with the advanced meta-experimental protocol of the transparent psi project procedures for testing the precognitive effect claimed by Bem
Source: PLoS One. 2025 Nov 5;20(11):e0335330. doi: 10.1371/journal.pone.0335330 (PMC12588471; doi:10.1371/journal.pone.0335330)
Supplement: Attachment — Submitted filename: Response to Reviewer.docx [file pone.0335330.s004.docx]

**Response to Reviewer 1**

**Review**

The manuscript, “Metascientific replication project with the advanced meta-experimental protocol of the transparent psi project procedures for testing the precognitive effect claimed by Bem,” investigates the replicability of claims about precognitive effects reported by Bem (2011) using advanced methodologies, including the AMP-TPP protocol, across three large-scale studies. While Study 2 replicated an exploratory finding suggesting a slight below-chance effect, neither the original precognitive effect nor the replicated anomaly was confirmed in Study 3. The results highlight methodological advancements for rigor in psychological research and suggest that observed results are better explained by method-derived anomalies rather than psi-theory.

The manuscript features a well-powered design and demonstrates commendable adherence to open-science and transparency practices, significantly enhancing its credibility and reproducibility while offering well-founded criticism of psi-theory. However, several areas warrant improvement, including clarifying key methodological details, improving the comprehensibility of the discussion, and addressing the results more thoroughly with critical consideration of specific psi concepts. Overall, this study is a valuable contribution to the field, and the authors’ commitment to rigorous research practices is evident. I believe that addressing the issues outlined above in a major revision would greatly enhance the manuscript’s quality.

For detailed comments, see below.

**Abstract**

The discussion section provides a commendable critique of the interpretation of psi-theory. However, the abstract could better reflect these critical insights. Currently, it presents a psi-derived anomaly (lines 43-44) as a plausible explanation for anomalous results, which risks implying equivalence with the much more scientifically grounded explanation of a method-derived anomaly. I recommend revising the abstract to highlight the significant limitations of psi-theory as an explanation, drawing on the critical insights from the discussion section.

To address this concern, the following sentence was added to the abstract (see lines 44-46): “Using conventional standards, based on the lack of replicability in Study 3 and absence of an accepted scientific theory, the second scenario appears more plausible.”

**Introduction**

1) Consider avoiding the introduction of abbreviations in headings, such as 'TPP' in '1.3.1. The Transparent Psi Project (TPP) Procedures.' Instead, use the full term, e.g., 'Procedures for the Transparent Psi Project,' and introduce the abbreviation in the main text of the section.

Yes. The introduction of the abbreviations is now limited to the main text

2) The manuscript employs the term “counterfactual” multiple times, but it may be unclear to readers who are not already familiar with its usage in this field. Although the preregistration provides clarification, the main text currently does not. Please offer a concise definition of “counterfactual” in the manuscript itself—ideally at first mention—to ensure all readers can fully grasp its relevance and application in the study.

Agreed. Section 1.3.2. now presents an introduction to the counterfactual test strategy of the AMP (at first mention). See lines 164-177: “For two examples see Section 1.4., where the AMP-based test strategy of counterfactual meta-experimentation (CFME) is described for capturing diagnostic information about the reliability of the scientific process that is used in this replication project. In general, counterfactual test strategies can empirically investigate the following question: What would have been the result if, for example, a different independent variable, or no independent variable, had been used as part of the employed scientific process? In the present work, as one type of counterfactual strategy, a random event generator (REG) substituted for the participants who are the source of the tested independent variable (see Section 1.4. for details). This counterfactual (REG-based) experiment should report non-significant results unless the scientific process were to produce systematic measurement errors. In short, the CFME-based test strategy seeks to reduce the risk of mistaking false-positive covariations—between outcome measure and the tested intervention—for true-causal ones by confirming empirically the absence of significant systematic error sources.”

3) Lines 132-133: The text states that the anomalous effect was observed only when erotic images were used as a reward, but it is unclear how these images functioned as such. Were they only revealed to participants upon correct guesses, or was there another mechanism at play?

Although this is explained later in the manuscript, it would greatly help readers to have a concise explanation of Bem’s original design and the reward structure at this point in the text. This also applies to the section covering lines 212–220.

A sentence was added to allow for a better understanding of this first, brief description of Bem’s original design (see lines 133-136): “Briefly, the reward procedure in Bem Experiment 1 [35] involved the presentation of either an erotic or non-erotic image following the selection by the participant of the correct side (left or right) on the computer screen, i.e., the side where the image will be presented (in the test for precognition).”

In addition, as was also requested in Comment 6, the relationships between moderating (reward), dependent, and independent, variables are described in new, revised form for clarity (see lines 230-237): “First, Fig 1A illustrates the experimental paradigm of Bem Experiment 1 [35]. In this setup, the assumed precognitive ability of participants is the independent variable. The dependent variable was defined as the percentage of correct predictions regarding the side (left or right) of a computer screen on which an image will occur (i.e., the successful guess rate). According to Bem’s interpretation of his observations, the nature of the image turned out to be a crucial moderating variable: only for erotic images the percentage of successful guesses differed significantly from mean chance expectancy [35]. No statistically significant deviation was, however, reported for trials that used non-erotic (neutral) images.”

4) Lines 166–171: The three classes of errors—class-A, class-B, and class-C—are presented in a rather abstract manner, making them difficult to understand without concrete examples. Additionally, the phrase “repeated performances” in the discussion of class-B errors is unclear; please clarify what exactly is meant by “performance” in this context. It would be beneficial to offer a clearer definition of each error category earlier in the manuscript, for instance in Section 1.3.2, so that readers are not left wondering about these distinctions until Section 2.2.2.

Good point. As requested, the corresponding paragraph in Section 1.3.2. was re-written to explain the three error types already at first mention (see lines 178-192): “The AMP-based approach, including the strategy of CFME, allows for the quantitative evaluation of the reliability of a research paradigm at a high level of detail; that is, the AMP can measure up to three different types of systematic error, called class-A, class-B, and class-C error in AMP terminology (see also Section 2.2.2.). A brief overview is provided next: (i) Systematic class-A error presents a bias in the overall scientific process—independent of applying control and experimental conditions. Examples of this type of error are intrinsic imbalances in the measurement procedure, including a potentially biased statistical analysis. (ii) Systematic class-B error presents bias as a function of repeated performances of control and experimental conditions. This type of systematic error might occur, for example, in studies where the same participant performs control or experimental sessions multiple times. For prior work assessing the presence of systematic class-B error in a study see ref. [25]. (iii) Systematic class-C error is bias due to any kind of uncontrolled performance of a control condition. An example of this type of error is a hidden effect of the control condition that competes with the effect of the experimental condition and thereby might produce a false-negative result.”

5) “For explanation, a hidden bias might produce a false-negative result because of a concealed shift of the measurement baseline as a function of a falsely reduced, or falsely increased, rate of successful guesses which might—in turn—prohibit the observation of a true-positive finding” (lines 197–200). This explanation is somewhat convoluted and unclear. What is meant by “a concealed shift of the measurement baseline”? I think the word baseline could be misleading in this context, as you refer to the chance level, if I am not mistaken. Maybe you can clarify this and make it a little bit more comprehensive.

It is agreed that the description using the term baseline is not helpful at this point in the text. Therefore, this passage was removed. The revised Section 1.3.2., which comes earlier in the manuscript, now already addresses how various biases might lead to false-positive or false-negative results. For examples, see lines 174-177 and 190-192.

6) The sentence “For a brief review, Bem [35] reported that a person’s precognitive ability (the independent variable) was revealed as an effect on the percentage of successful guesses in the selection of the correct target side (left or right) on a computer screen (the dependent variable) where an erotic image will be presented as a reward experience (the moderator variable).” (l. 212-216) is quite lengthy and hard to follow. You might consider revising it for brevity and clarity, ensuring each variable is clearly introduced and its role in the study is made more comprehensible.

As was already mentioned in response to Comment 3, the relationships between moderating (reward), dependent, and independent, variables are described in new, revised form for clarity. See lines 230-237.

7) Lines 222–225: This explanation of sham-erotic trials is unclear. If these trials involve no image being displayed, it is difficult to understand how a “successful” guess is determined and what happens in place of the erotic image. Please clarify what participants see on the screen during these sham-erotic trials. Again, I realize that you explain this in more detail later on, but as with previous comments, I believe it would help the reader to fully understand the sham conditions already at this stage.

Agreed. A sentence was added to correct for this (see lines 245-247): “More specifically, in the case of the sham-image trials, only a grey background image is revealed to the participant—irrespective of whether the guess by the participant was successful or not.”

8) Lines 229–248: This section provides a solid explanation of the control strategy for participants; however, it contains some redundancies and could benefit from improved language flow. I recommend revising this section to address these issues and enhance overall clarity and readability.

Agreed. This whole paragraph was revised accordingly (see lines 251-264): “Third, Fig 1C illustrates the CFME-based control strategy that removes the independent variable from the experimental process. This complies with the standard definition of a control condition, namely the assessment of the dependent variable in absence of the independent variable. As described before, in the present study, the independent variable is the psi-anomalous factor (i.e., precognitive ability) associated with the study participant. Consequently, the CFME-based design of this replication project included a comparative test strategy involving both experiments performed in the presence as well as in the absence of participants (using both erotic and non-erotic images). Specifically, for the participant-absent experiments, a random event generator (REG) substituted for the human operator in the performance of the “guessing” task. Here, the counterfactual (control) condition seeks an answer to the question: Can humans outperform the performance of a REG in predicting the correct target side on the computer screen? Such a control condition had neither been implemented in original Bem Experiment 1 [35] nor in subsequent replication experiments (e.g., [36]).”

As an additional consequence of this major revision, the legend to Fig. 2 was also revised to achieve better comprehensibility (see lines 266-270): “(A) In Bem Experiment 1, the source of the independent variable is the human operator (the participant) who presses one of two keys (A or L) on a keyboard—indicating the guess of the target side (the left or right curtain behind which the image is presumed to be hidden) on a computer screen. The target side and the type of target image (erotic or non-erotic) is determined using a REG.”

9) Lines 255–257: What is the specific advantage of using a Random Event Generator as a control variable? Wouldn’t a binomial test with p=0.5 effectively account for random selection, rendering the Random Event Generator unnecessary? Please clarify and emphasize the added value of simulating random choice with a machine compared to simply testing a binomial function.

This is, of course, an important question. The brief answer is that the AMP-based test strategy makes no assumption about whether the operationally-produced randomness, which is essential to Bem’s original design, matches the theoretical ideal of randomness. This is of particular importance when testing claimed results that challenge the known laws of physics; that is, no assumptions are made but instead the strategy is to empirically verify such basic assumptions (of randomness). A note was added to better point the reader’s thinking in this direction (see lines 276-279): “This assessment includes the detection of possible bias for outcome measures that solely rely on tests against the theoretical ideal of chance (e.g., see the unknown presence of a biased coin in coin flipping experiments).”

Additional revisions by authors:

Lines 296-298: The sentence was revised to read: “For one example where—using PCH—an unsuccessful confirmatory psi study was turned into an apparently successful psi study see ref. [22].”

**Methods**

10) Sample Size Estimation: The manuscript does not provide information on how the sample size was determined. While the preregistration includes a section describing Monte Carlo Simulation Studies, it does not specify the assumptions underlying these simulations. The preregistration also cites a preprint by Kekecs et al. (2019), where the sample size calculation is thoroughly described. However, to ensure readers are adequately informed, a brief explanation of the sample size justification should also be included in the main manuscript.

Additionally, the authors should provide more detail on the rationale for increasing the sample size from one experiment to the next. This explanation would help readers better understand the methodological decisions and their implications.

Thank you for pointing out this omission. In Section 1.6., we added “See Section 2.6.9 for the rationale of sample size determination.” See lines 313-314. We then added Section 2.6.9. (Determination of sample sizes and estimation of statistical power) at the end of the Method section. There, we explain in detail the rationale for the effect size estimate and sample size determination in the TPP project as well as the AMP-TPP studies, including the reason for increasing sample size in consecutive studies in order to ensure a power of > 0.95 of each replication study (see lines 678-695): “Overall, the AMP-TPP studies were designed to provide a power of 0.95, with the aim to keep the probability for alpha-error and beta-error balanced at 5%. Specifically, the pre-specified number of trials to be analyzed in Study 1 was adopted from the TPP study as outlined in Kekecs et al. [20]. There, the authors based their power and sample size calculations on an effect size estimate of a correct guess rate of 51%, taking into account that, although Bem’s original Experiment 1 yielded 53.1% successful guesses in trials involving erotic images, later meta-analyses indicated a lower effect. Simulations reported in Kekecs et al. [20] showed that—with a sample size of N = 37,836 trials—the probability of the TPP study to correctly reject the null hypothesis (if the successful guess rate is 51% or higher in the population) is 95% for simulations assuming no systematic individual differences between participants, and 90% for simulations assuming large personal differences. The number of trials in Study 2 was pre-specified at N = 127,000 trials based on power calculations for a one-sample binomial test and Monte Carlo simulations, assuming as an effect size a true successful guess rate of 49.48% (as indicated by the data from Study 1), and a desired power of > 0.95. The same procedure and rationale led to the specification of a sample size of N = 217,800 trials in Study 3. Here, an effect size of a true successful guess rate of 49.61% was assumed (as indicated by the weighted mean of the results found in Study 1 (49.48%) and Study 2 (49.65%)) and the desired power was again set at > 0.95.”

11) Please include details about the experimental software that was used in the experimental setup.

In Section 2.2.1., links were added to the online repositories where the code for the experimental software can be accessed. See lines 390-393.

12) Section 2.2.2: How does an S(O) trial differ from an S(X) trial? If I understood the sham trials correctly (and I am not entirely sure that I did), these trials seem to conclude without showing any picture (whether erotic or non-erotic) to the participant, even if they guessed the side of the image (or non-image?) correctly. However, this raises a question: how does the absence of an erotic picture differ from the absence of a non-erotic picture? Aren’t they effectively the same? While you address this later in the manuscript, a brief clarification earlier in the text would prevent readers from becoming confused or wondering about this distinction.

As a consequence of the reviewer’s Comments 12 to 15, Section 2.2.2. was re-organized and rewritten for better comprehension. This started with a new introduction to Section 2.2.2. (see lines 395-404): “The AMP-based test strategy distinguishes between so-called ‘true-experiments’ and ‘sham-experiments’ [25]. For explanation, in the context of the present investigation, true-experiments are called the experiments that are performed by the participants using either erotic or neutral images as moderating variables. By contrast, in the present study, sham-experiments (or sham-test conditions) are the experiments (or test conditions) that are conducted in one of the following counterfactual ways: (i) By the participants in the absence of a moderating variable (see Fig 1B), (ii) by a REG-based process which substitutes for the presence of the independent variable, i.e., the performance of the participant (see Figs 1C and 2B), and (iii) by a REG-based process in the absence of a moderating variable. For more detailed explanations see further below.”

We now also provide a more complete description and explanation of S(X)) and S(O) conditions (see lines 452-464): “Note that sham-erotic (S(X)) and sham-neutral (S(O)) image trials are identical, except with respect to the labelling of the data—unbeknownst to the participant—as sham-erotic or sham-neutral image trials. Again, when performing these sham-image trials, the participants received no reward; that is, only a grey background was displayed even though the participant had chosen the correct target side. Therefore, the participants thought that they had chosen incorrectly, even if they had been successful in choosing the correct target side as selected by the REG. In short, compared to the pure-session experiment (Fig 3A), the participants in the mixed-session experiment (Fig 3B) unavoidably experienced—on average—less positive feedback, i.e., the experience of a reward in the form of an erotic image upon guessing successfully. This constitutes a difference to the experimental designs implemented by Bem [35] and Kekecs et al. [20]. To be sure, note that the here-employed AMP-based experiments with sham-image trials only served the purpose of revealing possible systematic errors in association with the presence of the moderating variable as part of the scientific process.” Also, the legend to Fig 3 was revised accordingly (see lines 446-450): “(A) Standard experiment with the so-called ‘pure session’. Here, each participant performed 36 true-image trials with erotic (X) or neutral (O) images. (B) AMP-based experiments with the so-called ‘mixed session’. Here, each participant performed 18 true-image trials with erotic (X) or neutral (O) images, and 18 sham-image trials which are labelled S(X) or S(O). Left and right indicate the two sides of the computer screen (compare Fig 2).”

A new paragraph describing the (REG-based) sham-test condition without moderating variables in Study 1 was also added (see lines 473-478): “Finally, a third type of sham-test condition (S) was performed in Study 1, namely a test condition that removed both the moderator variable as well as the participant from the experimental process; again, here, REG-based (sham-)experiments were performed in the absence of the moderating variable. The sham-image trials that were performed by the REG are labelled S(X)_S_ in order to clearly distinguish them from the sham-image trials that were performed by participants which, again, are labelled S(X).”

13) Lines 404–412: The terms "X" and "O" are used in this section but are only explained later in the manuscript, and even then somewhat implicitly. To ensure that readers can fully understand the meaning of this nomenclature, consider providing a clear and concise explanation at the first mention of these terms. This will help avoid confusion and improve the overall clarity of the manuscript.

Agreed. The relevant terminology (X, O) is introduced in lines 414-421: “The terminology used in this AMP-TPP replication project, including the abbreviations for the different types of experiments (e.g., X, O, and S), is described next. The experimental condition (X) presents the condition where the participants perform the experiment with erotic images as the moderating variable. The control condition (O) presents the condition where the participants perform the experiment with neutral images as the moderating variable. Again, Bem (2011) reported that experiments with neutral images do not yield the claimed precognition effect; hence, these experiments with neutral images serve as a form of control experiment (O).”

Also, a new description of the sham-experimental versions of X and O was added, including the addition of new labels (X_S_ and O_S_) for better distinction. See lines 425-428: “Such REG-based sham-experiments, i.e., the experiments without participants, were systematically conducted for both the experimental condition (X) and the control condition (O), whereby—for easy distinction—the REG-based version of the experimental condition (X) is labelled X_S_ and the REG-based version of the control condition (O) is labelled O_S_.” As a consequence, the respective figure legends (see lines 754-756, 831-834, 872-873) were also revised. The labels in Figs. 5, 8, and 9, were also updated accordingly.

14) Lines 426–444: This section contains numerous redundancies and over-explains the concept of pure sessions and mixed sessions, with repeated references to Figure 3. To improve readability and streamline the text, consider consolidating the explanations and minimizing repetitive references to the figure, focusing on delivering the essential information succinctly.

Agreed. See the above revisions in response to Comments 12 and 13.

15) Lines 439–444: Assuming the existence of a psi-phenomenon or a similar concept, wouldn’t informing participants that their choice was wrong—when it was actually correct—risk inducing frustration? This could open the study to criticism from psi-supporters, who might argue that the presence of sham trials does more than control for the absence or presence of erotic image rewards; it could actively introduce frustration. A psi-defender might suggest that such frustration could interfere with psi ability. Would it not have been better to provide participants with feedback about whether their choices were correct or incorrect without displaying the image? This approach could control for reward absence while avoiding potential frustration effects.

This is a theoretically valid argument. In designing these sham trials it was, however, of primary importance that absolutely no difference in reward type exists between S(X) and S(O) trials, because they function as control trials for the test of the hypothesis brought forward by Bem (2011) which holds that the nature of the reward image is the moderator variable of the purported precognition effect. In other words, we cannot give differential feedback because we have no grounds on which we can assume with certainty that there is no differential effect of such differential feedback given to the participants, even if this feedback were to consist merely of the words “erotic” / “non-erotic” or “success”/”failure”.

This potential limitation should be addressed and discussed in the Discussion section.

In order to explicitly acknowledge this limitation already in the Methods Section, we added the following sentences (see lines 458-464): “In short, compared to the pure-session experiment (Fig 3A), the participants in the mixed-session experiment (Fig 3B) unavoidably experienced—on average—less positive feedback, i.e., the experience of a reward in the form of an erotic image upon guessing successfully. This constitutes a difference to the experimental designs implemented by Bem [35] and Kekecs et al. [20]. To be sure, note that the here-employed AMP-based experiments with sham-image trials only served the purpose of revealing possible systematic errors in association with the presence of the moderating variable as part of the scientific process.”

16) Lines 450–456: The detailed description of the consent procedure is overly lengthy and could be streamlined. Condensing this section would improve readability without compromising the essential information about the consent process.

We agree that this description of the consent procedure is rather lengthy. Originally this description was more concise. For explanation, in the course of the submission process it was, however, requested by the technical editors of PLOS ONE that we expand on the consent procedure. We therefore hesitate to shorten this section again now.

17) Lines 462–465: The number of participants is missing from this section.

We included the number of participants. See line 496.

Including study-wise reporting of participant demographics would enhance transparency and allow readers to better understand the sample characteristics for each study.

Sample characteristics were not significantly different between studies.

18) Section 2.6.1: It seems there were no exclusion criteria for data in the experiment. If that is the case, please state this explicitly to ensure clarity for the reader.

Section 2.6.1. was rewritten in the following way (see lines 531-537): “Incomplete sessions (< 36 trials) were included, to prevent optional stopping bias. Probably due to latency issues of the internet connection, on rare occasions sessions over-ran up to a maximum of 42 trials. This resulted in a very small proportion (< 0.05%) of unplanned extra trials. These trials were also included in the analysis. All trials up to the pre-specified sample size were included in the analysis (Study 1: N = 37,836 trials; Study 2: N = 127,000 trials; Study 3: N = 217,800 trials). All data in excess of the pre-specified sample size was excluded.”

19) Lines 496–497: The statement *“Data was pooled across participants for each experimental condition of each study”* is unclear. Please clarify what is meant by that statement.

We deleted this unclear statement since it was irrelevant for the topic “Inclusion and exclusion rules“.

20) Line 552: What does BUJ stand for?

Thanks for catching this, here information got lost formatting. Originally, the sentence read: “This knowledge-based prior was originally proposed by **B**em, **U**tts and **J**ohnson [40], and, thus, it is referred to as the BUJ prior.” We have restored this original version. See lines 588-589.

21) Line 588 (not line 558): You state, “Based on this, a bidirectional analysis was conducted (...).” Why is a bidirectional analysis conducted when the hypothesis being tested is unidirectional—that the hit rate is below 50%? Please clarify the reasoning behind this choice of analysis, as it seems inconsistent with the stated hypothesis.

Sections 2.6.5. and 2.6.6. describe the analysis that was performed as exploratory analysis in Study 1 and subsequently preregistered as confirmatory analysis for Study 2 and 3. As described in Section 2.6.5., this analysis tests a bidirectional hypothesis and thus the “definition of hypothesis confirmation is independent of the direction of the observed effect” (see lines 615-616). We have now added the following clarification of our reasoning at the end of Section 2.6.5. (lines 616-622): “The reason for choosing a bidirectional hypothesis—rather than a unidirectional hypothesis in the opposite direction—is that only within the framework of a bidirectional analysis could the observed results of Study 1 be interpreted as a replication of some kind of precognitive effect [35]. The precognitive effect would then be understood as an effect where the participants could both increase as well as decrease (e.g., in the form of a precognitive avoidance effect; see Section 4.3.) the frequency of erotic images being displayed to the participants.“

We also added the following clarification at the start of Section 2.6.6. (lines 625-631): “In two subsequent replications (Studies 2 and 3), it was explored whether or not the result of the exploratory analysis of Study 1 could be confirmed. To this end, Studies 2 and 3 tested—in a prospective confirmatory fashion—the hypothesis that a significantly higher or lower rate of successful guesses is observed than what is to be expected by chance. For this purpose, the same statistical test as mentioned above in Section 2.6.5. was preregistered to be applied to erotic as well as non-erotic, i.e., neutral, trials, as performed by human participants or a REG.“

22) Section 2.6.7: The procedures for Experiments 2 and 3 are heavily influenced by the results of Experiment 1, particularly as these results motivated the analyses to replicate a systematic deviation of the hit rate below 50%. Presenting the methods for all experiments in direct succession makes it challenging to follow the reasoning and motivations behind certain design choices, such as the analysis of temporal dynamics of hit rates described in lines 594–596. To improve clarity, I strongly recommend presenting the results of Experiment 1, followed by an intermediate (brief) discussion of Study 1, before introducing the methods for the subsequent experiments. This approach would help readers better understand the rationale for specific design and analysis choices.

We understand, and agree with, the observation that presenting the methods for all experiments in direct succession makes it challenging to follow the reasoning and motivations behind certain design choices. However, alternatively, since the complete presentation of the results requires ca. 3 pages and three different figures, the downside of presenting the results of Study 1 (and figures) directly after describing the methods of Study 1 would be a considerable disruption of the flow of the Method section. Weighing the pros and cons of either strategy, we decided to mention in the Method section only the key finding of Study 1 without any additional details, just so that the basic reasoning becomes understandable.

So, in the Method section one can now find the following sentences describing the reasoning and motivations behind choosing the bi-directional hypothesis (as was already partly reviewed in the responses to Comment 21):

Lines 602-606: “After analyzing the data collected in Study 1 according to the preregistered confirmatory analysis, a PCH-based exploratory analysis was also performed (see Section 1.5.). This investigation was motivated by the observation of a substantial deviation in the observed hit rate—compared to that expected on average by chance—in the direction opposite to the preregistered hypothesis.”.

Lines 614-622: “Note that because of the bidirectional nature of the analysis, this definition of hypothesis confirmation is independent of the direction of the observed effect. The reason for choosing a bidirectional hypothesis—rather than a unidirectional hypothesis in the opposite direction—is that only within the framework of a bidirectional analysis could the observed results of Study 1 be interpreted as a replication of some kind of precognitive effect [35]. The precognitive effect would then be understood as an effect where the participants could both increase as well as decrease (e.g., in the form of a precognitive avoidance effect; see Section 4.3.) the frequency of erotic images being displayed to the participants.“

Lines 625-631: “In two subsequent replications (Studies 2 and 3), it was explored whether or not the result of the exploratory analysis of Study 1 could be confirmed. To this end, Studies 2 and 3 tested—in a prospective confirmatory fashion—the hypothesis that a significantly higher or lower rate of successful guesses is observed than what is to be expected by chance. For this purpose, the same statistical test as mentioned above in Section 2.6.5. was preregistered to be applied to erotic as well as non-erotic, i.e., neutral, trials, as performed by human participants or a REG.“

Regarding the reasoning and motivation behind the analysis of temporal dynamics of hit rates, please see our response to your next comment:

23) Lines 594–605: The exploratory analysis of the temporal dynamics of hit rates is somewhat puzzling. It seems odd to describe the plot elements intended to visually investigate these dynamics (lines 599–605) without including such a plot in the manuscript. Additionally, it is unclear on what values the plotted 95% confidence intervals are based. Please revise this section to clarify exactly what is being plotted and consider including the relevant plot(s). If multiple plots were generated and could not fit within the manuscript, this should be explicitly stated in this section to prevent readers from wondering why these plots are absent.

We reported the exploratory analysis of the temporal dynamics of hit rates simply because it was mentioned in the preregistrations. We agree with the reviewer that it is potentially confusing for the reader to describe this exploratory analysis at length but not report any results. At the same time, including all the plots would be a distraction from the main important information and the paper is already quite long and complex. Therefore, we now decided to report in Section 2.6.8. (line 646) “Deviations from the preregistered analyses” the following paragraph (see lines 670-676): “Finally, the preregistrations mentioned a number of possible exploratory analyses for determining whether there existed any unusual features in the temporal dynamics of the rate of correct guesses. Initial inspection of the accordingly plotted data did not, however, reveal any consistent patterns or other noteworthy deviations from chance expectancy. Therefore, this line of investigation was not pursued further and—in order not to distract from the main confirmatory findings—the results from these exploratory investigations will not be reported in the present publication.”

24) Lines 632–633: The statement, “this reporting format was chosen because it is the most common in the field of psychology and parapsychology,” is debatable. While ±SE error bars may be common, confidence intervals (CIs) typically provide more information regarding the interpretability and significance of effects. I am a bit confused as to why you opted to depict the SE rather than the CI, although using SE is acceptable.

In the field of parapsychology “mean ± SE error bars” still appears to be the most widely used format and we wanted to make the results as accessible as possible to that particular community.

25) Line 655: The statement, *“All preregistered analyses, frequentist, and Bayesian confirmed model M₀,”* is problematic. Frequentist analyses cannot confirm M₀ in the way suggested here; they can only fail to reject the null hypothesis. In other words, the absence of evidence is not evidence of absence in the frequentist framework. Please revise this phrasing to reflect the limitations of frequentist analyses accurately. However, Bayesian analyses can indeed provide evidence supporting M₀, so this distinction should be made clear here and in other relevant sections of the manuscript.

Thanks for pointing this out. We have corrected the sentence to now read (lines 712-713): “All of the frequentist analyses failed to reject the null hypothesis and all preregistered Bayesian analyses confirmed model M0 (see also Section 2.6.2.).”

26) Line 669: Revise “The Bayes Factor (0, 1)” to ensure consistency with the earlier use of BF₀₁, which is more common and aligns with the manuscript’s established nomenclature. Please ensure this is consistent throughout the text.

The nomenclature was changed to “The Bayes Factor (BF01)”. See line 727.

27) Lines 687–689: You only provide a verbal description of finding null results in this section. To ensure transparency and completeness, please report the full results, including numerical values, directly in the text. Avoid relying on the figure caption for this information, as including it in the text section will enhance clarity and accessibility for readers.

Full results are now reported in this section (see lines 741-748): “In these experiments, the REG substituted for the human operator. Only null results were observed for all three REG-based experiments (see Fig 5): For the pure session: X = 49.98% ± 0.26 (p = 0.934); for the mixed session: X = 49.93% ± 0.26 (p = 0.774); S(X) = 50.16% ± 0.26 (p = 0.537). These experiments thus confirmed that systematic measurement biases were unlikely to produce false-negative (or false-positive) results with the frequentist analysis employed in participant-based Study 1, e.g., as a function of hidden bias towards lower-than-chance probabilities of guessing successfully the future random events…”.

Full results are also described for Study 2 now (see lines 813-815): “Equally, the statistical null result for neutral trials (O) in Study 1 (Fig 7A) was again observed in Study 2 (Fig 7B), that is, O = 49.83% ± 0.14 (p = 0.236).”

28) Lines 711–712: The phrasing “this apparent 0.5% effect” is unclear. Please rephrase to make it more comprehensive.

For better clarity, we have rewritten the relevant texts. Lines 771-773: “Here the exploratory analysis consisted in applying a two-sided PCH-based analysis to the observed data (see Section 2.6.5.).”

Lines 781-793: “The analysis of neutral trials (O) and sham-neutral trials (S(O)) showed statistical null results, both for the pure- and mixed-session experiments (compare Figs 6A and 6B). In addition, as shown in Fig 6B, the mixed-session experiment showed non-significant results for both erotic trials (X) and sham-erotic trials (S(X)). By contrast, the exploratory analysis of the data collected during Study 1 suggested a potential deviation effect of -0.52% for the pure-session experiment (see X in Fig 6A; p = 0.042). Note that a p-value produced by a post-hoc exploratory analysis cannot be safely interpreted to indicate the statistical significance of the associated deviation from mean chance expectancy that was observed in the data. Instead, only p-values derived from confirmatory analyses can be coherently interpreted in terms of statistical significance. Therefore, an apparently significant finding emerging from an exploratory or PCH-based analysis should undergo confirmatory testing before being reported in a publication. To this end, the subsequent Study 2 sought to further investigate this exploratory finding of Study 1 by a high-powered confirmatory replication.”

29) Lines 722–724: The statement “These null results seemed to confirm the finding reported by Bem [35] that neutral trials, i.e., trials with non-erotic images, do not lend themselves to precognition” is somewhat misleading, as your well-powered study also found no evidence of precognition with erotic images. Please rephrase this to emphasize the absence of any evidence for general precognition in this study, avoiding implications that precognition might still occur with erotic images.

We agree that this might be potentially misleading and therefore deleted the statement “These null results seemed to confirm the finding reported by Bem [35] that neutral trials, i.e., trials with non-erotic images, do not lend themselves to precognition.”

30) Lines 724–726: The statement *“found a null result”* is misleading, as it suggests an equivalence test was conducted. Since no equivalence test was performed, you can only state that no significant effect of precognition was found.

Agreed. The relevant statements were rewritten to read (lines 781-784): “The analysis of neutral trials (O) and sham-neutral trials (S(O)) showed statistical null results, both for the pure- and mixed-session experiments (compare Figs 6A and 6B). In addition, as shown in Fig 6B, the mixed-session experiment showed non-significant results for both erotic trials (X) and sham-erotic trials (S(X)).”

Note, again, that the wording of Section 3.1.3. was revised to make absolutely clear that even a p-value of < 0.05 cannot be interpreted as statistically significant if it results from an the exploratory analysis (see lines 786-792): “Note that a p-value produced by a post-hoc exploratory analysis cannot be safely interpreted to indicate the statistical significance of the associated deviation from mean chance expectancy that was observed in the data. Instead, only p-values derived from confirmatory analyses can be coherently interpreted in terms of statistical significance. Therefore, an apparently significant finding emerging from an exploratory or PCH-based analysis should undergo confirmatory testing before being reported in a publication.”

Additionally, “only for both erotic trials (X) and sham-erotic trials (S(X))” implies a significant effect for the mixed-neutral (O) condition, but Fig. 6 shows no significant p-value (p = 0.185). It seems this is inferred from the mixed-O error bar not crossing the 50% line. However, the error bar represents SE, not the 97.5%-CI, so this conclusion is incorrect. Please correct the statement or clarify what you mean by it. Also consider using proper CI representation instead of SE, as suggested earlier.

This is a misunderstanding due to the way we have written the sentence. The word “only” was there to emphasize that only null results were found, nothing else. (We did NOT mean to imply that null results were found only in these conditions but not in others). For clarity, the word “only” was now removed. See line 783.

**Discussion**

31) I may have missed it, but the Discussion section does not seem to explicitly address how the replicated effect in Study 2, which occurred in the *opposite direction* of the assumed precognition effect, could be interpreted as evidence for psi-based precognition. Why would participants be systematically wrong in predicting the appearance of erotic stimuli? Even setting aside the valid criticisms of psi-based explanations raised in the discussion, does this reversed effect provide any meaningful evidence for psi phenomena? This question warrants clarification.

Agreed. The following sentences were added to explain why a below-chance prediction could still be conceptualized as a precognitive effect, at least from the psi perspective (see lines 984-990): “Regarding the apparent effect reversal in Study 2, i.e., the observation of an anomalous, below-chance rate of successful guesses, this finding could, for example, potentially be indicative of a precognitive avoidance effect, where participants use their precognitive ability to avoid being shown an erotic image. In other words, such a psi-based interpretation might argue that the hypothesized precognitive ability can manifest both as precognitive avoidance and precognitive preference.”

32) The discussion effectively balances the rigorous scientific perspective with the psi-based perspective when interpreting the results. You also provide a thorough and commendable critique of psi-based approaches. However, the discussion is occasionally overly extensive and, at times, redundant. Certain sections are difficult to follow and could benefit from clarification (see specific suggestions below).

33) Line 899: “(e.g., [13, 42-47)” Missing closing square bracket in citation

Added. See line 960.

34) Line 925: The terms “trickster effect,” “psi-missing,” and “experimenter psi” are introduced without sufficient explanation, leaving readers unclear about how these concepts might account for the results within a psi framework. Currently, these terms are mentioned without connecting them meaningfully to the findings. A more thorough discussion is needed to explain how these ideas could be applied to interpret the results.

We feel that a thorough discussion of these concepts would be beyond the scope of the present paper. However, nevertheless, we fully agree that it is useful for the reader to get some idea about the general direction of the reasoning behind such psi concepts. Therefore, the following sentences were added or revised (see lines 992-1001): “For explanation, these concepts are often referred to in the parapsychological literature for the purpose of explaining the non-replicability of an original finding—using unproven psi-based assumptions. For example, the concept of ‘experimenter psi’ claims that the psi ability of the experimenter interferes with the experiment and—therefore—the true cause of an observed null result in a replication study is the experimenter’s own psi (e.g., [43,44]). No reliable evidence in support of this claim exists. For a critical discussion of such psi-based concepts in relation to the frequent non-replicability of results in psi research see Section 4.7. More generally, some special, not-yet identified, psi-conducive factor could have been missing in the present study and—therefore—the original psi effect of Bem [35] could not be replicated.”

35) Lines 925–927: The statement “some special, psi-conducive factor could have been missing in the present study and—therefore—the original psi effect of Bem [35] could not be replicated” does not address the surprising opposite direction of the effect observed in Studies 1 and 2. How would a psi-follower explain (their way out of) this reversed effect? If the failure to replicate Bem’s findings is attributed to a missing "psi-conducive" factor, this explanation does not account for why participants performed systematically worse than chance rather than at chance. If psi-proponents cannot provide a plausible explanation for this, it should be explicitly addressed, as it further reinforces criticisms that psi lacks a rigorous, falsifiable theoretical framework.

See the responses to Comments 31 and 34.

36) Lines 930–944: In this section, you discuss the high stakes of accepting psi explanations, noting the significant challenge they pose to conventional assumptions. Given these high stakes, one could argue for the adoption of a more rigorous alpha threshold in psi research. A false-positive result—such as the one likely observed in Study 2—has a much higher “cost” than a false-negative result. For instance, accepting a false-positive might mean we’d have to start firing physics professors en masse. Adopting a stricter alpha threshold, as suggested in [https://www.nature.com/articles/s41562-018-0311-x], could help mitigate such risks and improve the reliability of findings in this controversial area of research.

We agree and added the following sentence (see lines 1009-1010): “For example, a lower threshold for the acceptable alpha-error rate is often required in such cases before drawing conclusions.”

37) Lines 960–969: This section is convoluted and difficult to follow. Please clarify for better readability.

The section was completely rewritten. It now reads as follows (see lines 1036-1053): “In summary, the results presented here can theoretically be interpreted both in terms of a psi-based explanation as well as conventional explanation. The psi-based interpretation suffers from the fact that so far no empirically testable hypotheses have been derived from the ideas concerning psi-based (anomalous) explanations of non-replicability (see also Section 4.7.). Nevertheless, the psi-based proposition of an unknown or uncontrollable psi factor always remains a possibility as an “explanation”—even if only as part of a preferred philosophical position—for an anomalous result that can be confirmed only once. By contrast, the conventional interpretation of the results comes with the benefit of not requiring any additional assumptions, since the results can simply be seen as statistical artefacts, for example, due to conventional sampling error. At the same time, the magnitude of the deviation observed in Study 2 is such that it would be considered—by convention—a rather unlikely event to occur by chance alone. Other possible sources of experimental error or measurement bias are not obviously apparent and have to some extent been controlled for by the counterfactual (e.g., REG-based) test strategies in this metascientific replication project. Therefore, for the time being, the true source of the observed anomalous deviation from chance remains unidentified and, hence, the scientific status of the once-replicated anomalous effect observed in Study 2 will be characterized as an ‘unidentified laboratory phenomenon’, or ULP in short.”

38) Section 4.4: The heading *“On observed anomalous results in parapsychology as ULPs”* is somewhat unclear and took me a moment to understand that this section discusses how psi-ists interpret ULPs as psi-related phenomena. Consider changing the title to something like *“Psi-Explanation Attempts of ULPs”* for greater clarity.

We are in this section primarily aiming to make clear that the characterization as ULP is applicable not only to the result presented here but to all results presented in parapsychology so far, and that their interpretation, in terms of evidence for psi, would be premature. To make this point more clear, the heading was thus reworded to (see line 1054): “4.4. On classifying all existing results in parapsychology as ULPs”.

39) Fig. 10: I don’t think this figure is essential and could be removed.

We would like to leave the figure in, because it has been proven helpful for clarifications in discussions among colleagues. In particular, in the psi community, the distinction between “detection” and “identification” is often neglected (or conflated) in the arguments for the possible reality of psi effects. By providing an illustration (Step 1 to step 2), attention is drawn to this key difference which is so essential to all types of anomalies research.

40) Lines 1038–1044: The terms “Ψ-epistemic” and “Ψ-ontic interpretations” should be briefly explained for clarity. Additionally, it should be made explicit that these refer to a different kind of psi than the one assumed in precognition. Furthermore, the phrase “nonlocal ontology or quantum mechanics based on time-reversing, retrocausal ontologies” is difficult to understand for readers who are not familiar with quantum physics. Providing a brief and accessible explanation would greatly improve clarity.

Agreed. To address these concerns, the following sentences were added in the respective places.

Lines 1111-1119: “Briefly, the concept of quantum nonlocality refers to the long-confirmed observation of informational correlations between quantum events occurring at two distant physical locations. Remarkably, these long-distance (above-chance) informational correlations can exist in apparent contradiction to classical space-time constraints (e.g., in violation of the classical assumption of local realism). However, it is well-understood that proposals for explaining intentional, that is, non-random, psi phenomena based on nonlocality and nonlocal correlations are incompatible with the established requirements of quantum theory. The subsequent paragraph will explain this fundamental constraint in more detail.”

Lines 1133-1137: “Note that—in the context of quantum-theoretical interpretations—the terms ‘Ψ-epistemic’ and ‘Ψ-ontic’ do not refer to any psi-anomalous forms of cognition (i.e., parapsychology) but merely to the quantum-mechanical wave function Ψ which can be interpreted either epistemically or ontically in the respective interpretations of quantum mechanics (for further explanations see ref. [59]).”

Lines 1140-1145: “Finally, again, the non-signalling principle equally applies to any form of time-reversing (retrocausal) quantum interpretation. That is, the non-signalling principle prevents the (quantum) transmission of signals or messages from the future to the present or past [e.g., (56)]. This well-known constraint on transmission directly challenges the plausibility of prior work claiming that quantum theory might explain the anomalous phenomenon of precognition [e.g., (60,61)].”

Lines 1477-1483: The above-mentioned new references [60,61] were added to the reference section.

41) Lines 1069–1072: Use a consistent enumeration style throughout the manuscript. For example, replace the (1), (2), ... style in this section with the previously used (i), (ii), ... style.

Done.

42) Section 4.6: This section feels somewhat redundant compared to other sections. Consider removing it and, if necessary, condensing its key points and integrating them into other relevant parts of the manuscript.

We agree that there is a degree of redundancy here, because many of the topics have already been mentioned earlier in the text. If you feel strongly about it, we can remove this section. Our preference would be to keep it, though. For some background, parapsychologist have long compared their efforts in anomalous cognition research to anomalies research in conventional science, in particular cosmology and high-energy physics. Some parapsychologists have insisted that the challenges are, at least conceptually speaking, comparable and, therefore, conventional (mainstream) scientists should keep an open mind about the possibility of psi. However, as Section 4.6. points out, there are clear differences between anomalies research in the physical sciences versus anomalies research in the cognitive sciences, namely for the latter, (i) the lack of a (psi) theory and (ii) the non-replicability of results. In short, these are the basic reasons why parapsychology is widely seen as pseudoscience (and not due to the mere fact that it is anomalies research). Again, these differences, to us, seem underrecognized in the psi-researcher’s community and hopefully will be better recognized by elevating these comparisons to the level of a section in the Discussion. To make this point more explicit, the title of Section 4.6. was changed to “Comparing psi research with anomalies research in physics” (see line 1176). Finally, in relation to your Comment 45, Section 4.6. also points out that psi research is widely regarded as (debunked) “pseudoscience”.

43) Lines 1110-1111: “In the history of modern science, there has been no other field of research that was able to maintain interest for so long as parapsychology” [underscoring added by the Reviewer for emphasis]. Is there literature supporting this claim? If so, please provide appropriate citations. Otherwise, consider softening the statement.

We agree that this manuscript is not the place for discussing the historical significance of parapsychology within the framework of modern science. Therefore, we have deleted the relevant sentences. In addition, the paragraph following these deletions was strongly revised, to provide clearer context for the introduction of the concept of anomalous non-replicability (ANR). See lines 1210-1222: “For background, some psi-researchers have argued for the view that (i) exploratory (PCH-based) methods enable more frequent observations of psi effects and (ii) the observed non-replicability under confirmatory conditions is itself a psi-derived anomaly; hence, in that view, the observed non-replicability is not the result of conventional factors, such as HARKing [67], confirmation bias [8], or intrinsic measurement bias (e.g., [25]), but is a psi phenomenon itself. For example, the frequent observation that an original finding cannot subsequently be replicated has also become known as the ‘Decline Effect’—occasionally implying that the observed non-replicability has an anomalous dimension also (e.g., [68,69]). Other unconventional (psi) ideas to explain the lack of replicability include the already-mentioned concepts of “trickster effect”, “psi-missing”, or “experimenter psi” (e.g., [13,42-45]). In summary, all these various proposals—in essence—argue that in the area of psi-research, the non-replicability of a finding does not necessarily indicate that this initial finding was a false positive. That position is here referred to as ANR or the ANR-hypothesis.”

44) Table 1: I think this table would fit better in the results section.

The table repeats the information about the present study that was already presented in the result section because it is supposed to help contextualize these results in the discussion section by comparing them to the original results by Bem (2011) and the results of the independent replication study by Kekecs et al. (2023). (We did not want to start a comparative discussion of our results versus independent results already in the Results section.)

45) A somewhat sacrilegious and deliberately provocative question: Psi research has been thoroughly debunked many times over, and rightly so—it’s a discredited field that has repeatedly failed to demonstrate credibility. Given this, why is it still necessary to invest significant effort and resources into debunking it yet again? Couldn’t this field simply be ignored as an irrelevant pursuit by a small fringe group of researchers, allowing science to focus on more pressing and meaningful topics? While I recognize that such questions might be reductive, some readers may share this perspective. Perhaps you could explicitly address these concerns in the manuscript and respond to them and also highlight how your study contributes to broader scientific and methodological progress, demonstrating its relevance beyond merely revisiting psi research.

We can empathize with this perspective to a large extent. Preregistered confirmatory psi-studies have produced almost exclusively null results, so one cannot blame anyone for losing interest in the field altogether. To be fair, though, Experiment #1 of Bem (2011) had not been tested under such rigorous conditions until the TPP (Transparent Psi Project; Kekecs et al., 2023)) embarked to do so, while at the same time producing a new benchmark for best practice in open science. We felt that it was appropriate to complement that effort by adding the AMP methodology (Walleczek and von Stillfried, 2019) to check for the possible presence of intrinsic systematic errors. This combined research effort resulted in one of the most methodologically-advanced replication projects (using the combined AMP-TPP research design).

As you have suggested, we have added some text to underline our belief that such improved (AMP-based) control conditions can also be of value to other research areas. We added to Section 4.1. the sentence (lines 919-921): “The use of this advanced methodological procedure is of course not limited to the field of parapsychology but instead can increase reliability of results in all research areas.”

In Section 4.8., we also expanded a sentence accordingly (see lines 1269-1272): “In the future, the adoption of such advanced research designs, including the use of CFME-based approaches for improving the detection of intrinsic measurement biases, might reduce the risk of mistaking false-positive for true-positive results not only in parapsychological research, but also in other research areas.”

These new statements are in addition to the two sentences referring to the aims of the present project at the end of Section 1. (see lines 79-81): “Therefore, one major aim of this replication project was to demonstrate how advanced control test strategies can improve the reliability of findings, not only for psi research, but frontier science in general”, and at the end of Section 1.6. (see lines 323-326): “In summary, this scientific investigation of the scientific process—as used in pursuit of a claimed psi effect—yields new insights into (i) the replicability of an apparent psi-anomalous result and (ii) how advanced control test strategies can help improve the credibility, not only of psi research, but any type of weak-effects experiments probing the frontiers of science. “

Finally, the concluding sentence of the abstract was also revised to make this point more explicit (see lines 47-49): “This project shows how rigor-enhancing test strategies can improve the reliability, not only of psi research, but any type of weak-effects experiments, including in psychology.”
