# Supplementary material for: Metascientific replication project with the advanced meta-experimental protocol of the transparent psi project procedures for testing the precognitive effect claimed by Bem
Source: PLoS One. 2025 Nov 5;20(11):e0335330. doi: 10.1371/journal.pone.0335330 (PMC12588471; doi:10.1371/journal.pone.0335330)
Supplement: Attachment — Submitted filename: Response to Reviewers.docx [file pone.0335330.s005.docx]

**Response to Reviewers (2 and 3)**

**Response to Reviewer 2**

**Response to Major Comment No. 1 (“Justification”):**

The reviewer is concerned about our portrayal of the basic rationale or justification for performing a replication study of the precognitive effect claimed by Bem (2011) [35]. In particular, the concern is expressed that without a substantial (re)analysis of the prior meta-analysis of 90 replication studies (Bem et al., 2016 [36]) our rationale for performing our metascientific replication project is either weak or unconvincing (“insufficient”).

**Response:** Thank you for pointing out that the rationale for performing a replication study of Bem Experiment 1 might be misconceived by readers. For sure, we did not want to give the impression that this work was motivated by a critique of the database used in the meta-analysis of Bem et al. (2016) [36].

Instead, this AMP-based replication project was motivated by the availability of the TPP procedures for testing the claimed precognitive effect by Bem (see Kekecs et al., 2023 [20]). Crucially, these TPP procedures were consensus-designed with the participation of Bem himself (see also Kekecs et al., 2022 [37]). This presented a rare instance in psi research where the author of an original psi study was involved in the design of an “independent” replication study of his/her work.

Therefore, the implementation of the consensus-designed TPP procedures in this AMP-TPP replication project allowed for a further assessment of the replicability of the precognitive effect claimed by Bem (see also Section 1.3.1.); again, Bem himself supported the use of the TPP procedures in the attempt to replicate his original findings.

To better prevent the possible misconception of the rationale (justification) for having performed the present replication project, the misleading sentence referring to the meta-analysis by Bem et al. (2016) was removed from the text. In addition, the immediately preceding sentence was revised to read:

Lines 123-125: “The present work investigated the most-widely discussed claim for an experimental psi effect in recent years, namely the precognitive effect claimed with Experiment 1 in the reports by Bem [35] and Bem et al. [36].”

Furthermore, in the Introduction (1.3.1.), the following sentence was revised to read:

Lines 141-144: “The implementation of these procedures was based on the transparent psi project (TPP) procedures for testing the precognitive effect claimed by Bem which were developed using a consensus-design process involving both sceptics as well as proponents of psi research, including Bem himself [37].”

In addition, the following sentences were added to provide further clarification of the rationale or justification for performing the present replication project with the TPP procedures.

Lines 144-148: “Again, this replication project sought to reproduce the consensus-designed TPP procedures which had previously been employed in a multi-laboratory replication project of Bem Experiment 1 [20]. In this way, a research protocol could be implemented in the present replication project that was widely supported by both skeptics and proponents of psi research.”

Finally, we agree, of course, that it would be a highly valuable effort to reanalyze the database used in the meta-analysis by Bem et al. (2016) [36] in terms of “… preregistration status, sample size adequacy, blinding, and handling of exploratory analyses”. But this would be an additional research project beyond the scope of the present experimental work, which was motivated by the question whether the precognitive effect claimed by Bem could be replicated with the consensus-designed TPP procedures (Kekecs et al., 2022 [37], 2023 [20]). Please note also the title of this manuscript.

**Response to Major Comment No. 2 (“Generalizability”):**

The reviewer is concerned about the (claim of) generalizability of the results of this metascientific replication project. Specifically, the reviewer writes that “… the replicability of the authors’ findings—particularly the anomalous below-chance effect observed in Study 2—requires verification by independent laboratories using the same experimental protocol, population characteristics, and analytical methods.”

**Response**: We agree. For brief review, (i) Study 3 conducted a high-powered replication attempt of the results from Study 2, and (ii) the results of Study 2 were found to be non-replicable in Study 3. As the reviewer has correctly suggested (i.e., the argument against the generalizability of our results), it would be necessary to perform independent replication attempts (by other laboratories) of the results of Study 2. Would other laboratories also find null results, as we did in Study 3, or – instead – significant results as we found with Study 2? Only further research will be able to tell.

Importantly, the Table in the Discussion lists all presently available replication studies of Bem Experiment 1 using the consensus-designed TPP procedures (Kekecs et al., 2023 [20], 2022 [37]). In short, so far at least, both the studies performed (i) in university settings using a multi-laboratory replication design (see Kekecs et al., 2023 [20]) as well as the online studies (our work) found that the original precognitive effect claimed by Bem was found to be non-replicable with the consensus-designed TPP procedures.

However, regarding the positive effect found with AMP-TPP Study 2, which was in the opposite direction (of the original precognition effect), we agree with the reviewer that only future independent replication studies can clarify whether this effect might be replicable (or not). Obviously, again, when we (the authors) attempted to replicate this apparent effect with the high-powered AMP-TPP Study 3, we failed to replicate our own prior positive finding (using the same protocol, near-identical population characteristics, and the same analytic methods).

To acknowledge that any final conclusions (re: the generalizability of our results) are impossible to reach with the present project, we had written Sections 4.4. (ULP) and 4.7. (ANR) in the Discussion. There, we sought to amply acknowledge that only future research will be able to clarify what the true nature is of the positive finding observed with Study 2: (1) either a psi-derived or method-derived anomaly (see Sect. 4.4.), including (2) as a non-replicable finding in the sense of either an anomalous (psi-derived anomaly) or conventional (method-derived anomaly) non-replicable result (see Sect. 4.7.).

In summary, we agree that only independent replication studies might clarify whether the positive result observed in Study 2 is replicable (or not). To emphasize this point even more, we added the following sentence in (final) Section 4.8. (Conclusions and outlook).

Lines 1284-1288: “To clarify whether the below-chance effect observed in confirmatory Study 2 (Section 3.2.1.), which was not found to replicable in Study 3 (Section 3.3.), might nevertheless be confirmed with independent replication studies, a multi-laboratory research effort could be conducted regarding this unexpected finding (see also further below regarding the topic of future research).”

**Responses to Minor Comments:**

**The reviewer writes**: “While the statistical analyses are well-described, the rationale for choosing specific priors in the Bayesian proportion tests (e.g., BUJ prior, replication prior) could be clearer. The authors should briefly explain why these priors were selected and how they influence the interpretation of the Bayes factors, particularly for readers unfamiliar with Bayesian methods in parapsychology.”

**Response**: In the AMP-TPP replication project, these specific statistical analyses were preregistered because the aim of this project was to reproduce, as closely as possible, the transparent psi project (TPP) procedures for testing the precognitive effect claimed by Bem (see Ref. [20]: Kekecs Z, Palfi B, Szaszi B, Szecsi P, Zrubka M, Kovacs M, et al. Raising the value of research studies in psychological science by increasing the credibility of research reports: The transparent psi project. Royal Soc Open Sci. 2023;10(2): 191375.) Since, in the TPP Project, the choice of these specific priors was the result of a consensus-design process, we prefer to quote (in Section 2.6.4.) only what the published Registered Report (Kekecs et al., 2023 [20]) says about this, including the TPP Project’s reference for the source of the BUJ prior (Ref. [40]: Bem DJ, Utts J, Johnson WO. Must psychologists change the way they analyze their data? J Pers Soc Psychol. 2011;101(4): 716-9.) without, however, adding any further thoughts and interpretations.

**The reviewer writes**: “Please make raw data and code publicly available. There are no data and code archived in the repository provided by the authors (<https://osf.io/8jteq/>)”.

**Response**: We thank the reviewer for alerting us to this issue. Apparently, due to a change of the OSF website architecture, the files uploaded to <https://osf.io/8jteq/> have become somewhat difficult to find. On <https://osf.io/8jteq/> click on either "Data" or "Analysis code" in the section called "Components". This then takes you to different webpages, namely <https://osf.io/68wy2/> (for data) and <https://osf.io/k5fnx/> (for analysis code). On these pages, clicking on "files" in the upper horizontal menu bar will bring you to <https://osf.io/68wy2/files/osfstorage> (for data) and <https://osf.io/k5fnx/files/osfstorage> (for analysis code). There, the respective files can be viewed/downloaded. As a consequence of this difficulty, we have revised the manuscript as follows:

Lines 342-345: For easy access to all primary data and analysis codes for the AMP-TPP metascientific replication project, including access to Github repositories, the following OSF repository was created: https://osf.io/8jteq/. There, all data (https://osf.io/68wy2/files/osfstorage) and analysis codes (https://osf.io/k5fnx/files/osfstorage) can be viewed.

Finally, we thank the reviewer for the thoughtful and constructive comments and criticisms. We hope that we were able to sufficiently address these concerns. For sure, the revisions resulting from the reviewer’s criticisms have helped improve the overall comprehensibility of the manuscript.

**Response to Reviewer 3**

The reviewer lists four minor issues requiring additional clarification.

**Response to Minor Issue No. 1:**

Regarding the Methods section, the reviewer is concerned that “the specific content” of the “minor technical adjustments” that “were made to the software” are not adequately described. The reviewer requires a further explanation.

**Response:**

For review, the present manuscripts reads (see lines 384-389): “Only minor technical adjustments were made to the software used by Kekecs et al. [20] to allow the online implementation, but the user interface was kept identical. As a consequence of the online setting, there was no personal briefing of participants or Q&A before or after the session. All instructions were instead given in written form via the experimental software. Participants were given the option to contact experimenters via email after the experiment.”

In response to the reviewer’s concern, we added a sentence emphasizing that the only changes made to the software code resulted from the adaptation of its use to online settings (see lines 389-392).

Lines 389-392: “In short, the changes made to the software code—as originally used by Kekecs et al. [20]—were exclusively a function of adapting its use to online settings where, unlike in university (laboratory) settings, no experimenters were present (for access to the used software code follow the online links below).”

**Additional note to the reviewer**: As is already described in the manuscript (see lines 944-946), just like the prior multi-laboratory replication project performed in university (laboratory) settings, the present online replication project also was unable to confirm the precognitive effect claimed by Bem.

Lines 944-946: “Note that for these overall null results it did not matter whether participants were performing the guessing task in a university setting [20] or online, e.g., in the comfort of their home (as in the present work).”

**Response to Minor Issue No. 2:**

Regarding the Discussion section, the reviewer is concerned about two different items and writes:

**Item 1**: “Sections 4.3-4.7 show a slight imbalance in the comparison between the psi interpretation and the traditional interpretation. Some paragraphs are piled with technical jargon, resulting in insufficient readability. It is recommended to use a table to compare the core viewpoints, evidential support, and limitations of the two interpretations, so as to enhance visualization.”

**Item 2**: “Simplify the discussion on the quantum mechanics part, focus on the contradiction between the "non-signal transmission principle" and the precognitive effect, and avoid deviating from the theme.”

**Response:**

Regarding **item 1**, we do certainly appreciate that given the highly-interdisciplinary research topic which is here investigated, the (sometimes difficult to understand) technical terms from several different fields of study are necessarily referred to in the Discussion. This includes some of the standard technical terms used in (i) parapsychology, (ii) philosophy of science, (iii) the field of metascience, (iv) anomalies research, and (v) fundamental physical theory, such as quantum theory. We believe that constructing a Table for providing a visualization of these concepts, terms, and interpretations, including various pros and cons associated with these concepts, terms, and interpretations as well as their possible inter-relationships, would be a highly-complex visual scheme. We prefer to keep a step-by-step conceptual discussion of terms. Again, our discussion attempted to provide some overview of frequently-used concepts from these fields of study by composing five consecutive sections (4.3. to 4.7.) with the aim of introducing these (technical) terms and their possible inter-relationships. In addition, in two of the Sections, we have introduced and explained in detail two new concepts of great importance to psi research, namely ULP (Section 4.4.) and ANR (Section 4.7.). Again, presently, in discussions of the field of parapsychology, all these different dimensions are highly relevant (4.3. to 4.7.). Therefore, in the Discussion section (4.3. to 4.8.), we sought to offer some relevant background for readers who might wish to start probing these challenging technical aspects at a deeper level. In short, we were hoping to give some initial guidance and direction for enabling further inquiries by interested readers (including based on the cited references).

Regarding **item 2**, we should acknowledge that another (previous) reviewer had asked us to expand on the description of this Section (4.5.) so that the relevant terms can be better followed throughout this high-level discussion of the possible significance of quantum theory in the interpretation of psi phenomena. Therefore, we do not want to reduce/simplify again these recently expanded passages in Section 4.5. Also, given that the significance of the "non-signal transmission principle" has been misunderstood so many times in the psi-based literature, we believe that it is highly relevant to provide a higher-level, historical context and introductory technical explanations (Section 4.5.) for that particular community.

**Response to Minor Comment No. 3:**

The reviewer has requested to “… add specific suggestions for future research to enhance the forward-looking nature of the study” (in Section 4.8., Conclusions).

**Response:**

We agree. Therefore, we have added the following sentence to the Conclusions (Section 4.8.).

Lines 1284-1288: “To clarify whether the below-chance effect observed in confirmatory Study 2 (Section 3.2.1.), which was not found to replicable in Study 3 (Section 3.3.), might nevertheless be confirmed with independent replication studies, a multi-laboratory research effort could be conducted regarding this unexpected finding (see also further below regarding the topic of future research).”

**Response to Minor Comment No. 4:**

The reviewer has requested to “Please supplement the limitations of this study.”

**Response:**

We are sorry if our efforts to portray the limitations of this study have not met the reviewer’s expectations. For explanation, our aim was to provide ample discussion of the limitations of the present replication project starting with the following questions (see lines 985-987): “What are important limitations of the present replication project? What important questions remain unanswered?” At least from our perspective, what follows on the next pages (and Sections), including the final Section (4.8.) on “Conclusions and outlook”, is a detailed discussion of the limitations of this replication project.

For example, to acknowledge the limitation that no final conclusions (re: the generalizability of our results) can be reached with the present project, we had also written Sections 4.4. (ULP) and 4.7. (ANR) in the Discussion. There, we sought to amply acknowledge that only future research will be able to clarify what the true nature is of the positive finding observed with Study 2: (1) either a psi-derived or method-derived anomaly (see Sect. 4.4.), including (2) as a non-replicable finding in the sense of either an anomalous (psi-derived anomaly) or conventional (method-derived anomaly) non-replicable result (see Sect. 4.7.).

Other examples are from Section 4.4. which directly acknowledges that, given the limitations of this replication project, the positive result observed in Study 2 can only be described as an “unidentified laboratory phenomenon” or ULP. Specifically, we have also offered these conclusions concerning limitations (see lines 1083-1086): “In summary, until the time when the true source of an apparent psi-anomalous phenomenon can be identified in a reliable manner, its scientific status is that of an ULP—instead of, prematurely, a psi-derived anomaly, i.e., a true-positive result (Fig 10).“, and, finally, in the Conclusions and outlook (Section 4.8., lines 1306-1310) “… that is, unless reliable evidence in support of the ANR-hypothesis could be obtained, research on anomalous cognition will likely remain an entirely exploratory program—with no prospect of becoming a recognized science capable of identifying ULPs as psi-derived anomalies using confirmatory protocols.”

Again, we are sorry if the reviewer is not satisfied with the given level of detail concerning the limitations of this replication project. Please indicate which relevant limitation(s) must be added to the Discussion section.

Finally, we thank the reviewer for raising possible concerns regarding the above issues. We hope that the resulting text revisions have largely addressed these concerns. Many thanks, again.
